# Factors associated with viral non-suppression among adolescents living with HIV in Cambodia: a cross-sectional study

**DOI:** 10.1186/s12981-018-0205-z

**Published:** 2018-11-17

**Authors:** Kolab Chhim, Gitau Mburu, Sovannary Tuot, Ratana Sopha, Vohith Khol, Pheak Chhoun, Siyan Yi

**Affiliations:** 1 KHANA Center for Population Health Research, No. 33, Street 71, Phnom Penh, Cambodia; 2 Division of Health Research, Lancaster University, Lancaster, UK; 3 National Center for HIV/AIDS, Dermatology and STD, Phnom Penh, Cambodia; 4 Saw Swee Hock School of Public Health, National University of Singapore and National University Health System, Singapore, Singapore; 5 Center for Global Health Research, Touro University California, Vallejo, CA USA

**Keywords:** Adolescents, Antiretroviral therapy (ART), HIV care and treatment, Viral suppression, Social determinants, Cambodia

## Abstract

**Background:**

Adolescents living with HIV on antiretroviral therapy (ART) have worse treatment adherence, viral suppression, and mortality rates compared to adults. This study investigated factors associated with viral non-suppression among adolescents living with HIV in Cambodia.

**Methods:**

A cross-sectional study was conducted in August 2016 among 328 adolescents living with HIV aged 15–17 years who were randomly selected from 11 ART clinics in the capital city of Phnom Penh and 10 other provinces. Clinical and immunological data, including CD4 count and viral load, were obtained from medical records at ART clinics. Adolescents were categorized as having achieved viral suppression if their latest viral load count was < 1000 ribonucleic acid (RNA) copies/mL. Multivariate logistic regression analysis was performed to identify factors independently associated with viral non-suppression.

**Results:**

The mean age of the participants was 15.9 years (SD = 0.8), and 48.5% were female. Median duration on ART was 8.6 (interquartile range = 6.0–10.6) years. Of total, 76.8% of the participants had achieved viral suppression. After adjustment for other covariates, the likelihood of having viral non-suppression remained significantly lower among adolescents who were: older/aged 17 (AOR = 0.46, 95% CI 0.21–0.98), had been on ART for more than 9 years (AOR = 0.35, 95% CI 0.19–0.64), had most recent CD4 count of > 672 (AOR = 0.47, 95% CI 0.26–0.86), had a relative as the main daily caregiver (AOR = 0.37, 95% CI 0.17–0.80), and did not believe that there is a cure for AIDS (AOR = 0.40, 95% CI 0.21–0.75) compared to their reference group. The likelihood of having viral non-suppression also remained significantly higher among adolescents who had first viral load > 628 RNA copies/mL (AOR = 1.81, 95% CI 1.05–4.08) and among those who were receiving HIV care and treatment from an adult clinic (AOR = 2.95, 95% CI 1.56–5.59).

**Conclusions:**

The proportion of adolescents living with HIV with viral suppression in this study was relatively high at 76.8%, but falls short of the global target of 90%. Programs targeting younger adolescents and adolescents in transition from pediatric to adult care with a range of interventions including psychosocial support and treatment literacy could further improve viral suppression outcomes.

## Background

Human immunodeficiency virus (HIV) is a leading cause of global burden of disease [1]. In recent years, significant progress has been made in increasing access to antiretroviral therapy (ART) for people living with HIV [2]. A key goal of ART is to suppress the replication of the virus. Suppressed viral replication facilitates restoration of the immune function and significantly reduces the risk of onward HIV transmission [3]. The World Health Organization (WHO) introduced viral load monitoring as a gold standard to follow up the treatment effectiveness in 2013 [4], and Cambodia adopted these guidelines in late 2015 [5]. The Cambodian national guidelines recommend that viral load testing be performed 6 months after ART initiation and repeated routinely once a year.

Despite the increasing access to ART, numerous studies have demonstrated suboptimal levels of viral suppression in different populations in many low-resource settings [1]. In particular, adolescents living with HIV tend to experience worse immunological and viral suppression outcomes compared to adults [6–9], which in turn contributes to their higher mortality [10, 11]. Indeed, HIV‐related deaths among adolescents have tripled since 2000, making HIV the second leading cause of death among adolescents globally [12]. While health related data for this age group remain scarce, there is emerging evidence indicating that adolescents living with HIV have been facing challenges in accessing HIV services [13].

Studies from different settings have identified a wide range of factors that may determine HIV treatment outcomes among adolescents. These factors include sub-optimal disclosure of adolescents’ HIV status [14, 15], poor linkage to care [10], attrition from care and treatment [16], poor transition from pediatric to adult services [17], challenges with adherence to ART [18], economic hardships [19, 20], and AIDS-related stigma [15, 21]. These factors commonly prevent attainment of optimal ART outcomes among this young population.

In Cambodia, 8512 children and adolescents are living with HIV and accessing care and treatment services from 37 ART clinics across the country [22]. Majority of them are infected through mother-to-child transmission [5]. According to the national guidelines, children and adolescents up to the age of 15 are recommended to receive HIV care and treatment services from pediatric ART clinics and to thereafter be transitioned to adult clinics. However, in practice, most adolescents living with HIV transition to adult care between 15 and 20 years of age [5]. Previous studies have indicated that, similar to the global situation, adolescents living with HIV in Cambodia face challenges related to transitioning into adult services [23], AIDS-related stigma and issues of confidentiality [24, 25], all of which affect HIV treatment outcomes. In addition, there is very limited information about viral suppression and its determinants among adolescents living with HIV in Cambodia.

Given the above concerns and data gaps, the objective of this study is to determine the prevalence rate of viral suppression and factors associated with viral non-suppression among adolescents living with HIV in Cambodia. This information will be critical in identifying program interventions that can be further strengthened to optimize ART outcomes among this population in Cambodia and other resource-limited settings.

## Methods

### Study design and population

This cross-sectional study was conducted in August 2016 as part of a larger study exploring challenges in transition into adult care among adolescents living with HIV in the capital city of Phnom Penh and 10 other provinces. Details of this study have been reported elsewhere [20, 26]. A structured questionnaire was used for face-to-face interviews complemented by routine clinical data retrieved from medical records in ART clinics. The study participants included adolescents living with HIV aged 15–17, who were receiving HIV care and treatment services from 11 ART clinics. In 2016, the number of adolescents living with HIV in 18 clinics represented approximately 90% of the total number of adolescents living with HIV in the country. The remaining 10% were distributed across 21 sites, each with less than 10 adolescents.

Participants were selected in three stages. First, a two-stage cluster sampling method was used to select clusters to sample participants from the selected clinics, using a methodology developed by the WHO [27]. A comprehensive list of all ART clinics in the country with adolescents living with HIV population estimates was created and used to select the clusters after excluding ART clinics with less than 10 adolescents. This left a universe of 598 adolescents receiving care and treatment from the 18 major ART clinics. To minimize costs of data collection, additional seven clinics with less than 20 adolescent clients aged 15–17 were excluded.

Second, the required sample size was determined taking into account population size, confidence interval, and estimated proportion of the population with the outcome of interest [28], which in this case is viral suppression. Assuming that 50% of adolescents had viral suppression, a finite population size of 598, a design effect of 1.4, and a 10% dropout or refusal rate, a minimum sample size of 310 was required for the study to measure variables among selected adolescents with a confidence interval of 4.3%.

Third, the number of participants recruited from each cluster was determined. Because the size of the ART clinics varies, the probability proportional to size sampling method [28] was used in order to ensure that adolescents in larger sites have the same probability of being included in the sample as those in smaller sites. Cumulative population size of each ART clinic was calculated, sampling interval was determined, and potential participants were randomly selected from the database using a random number table.

Finally, a list of selected adolescents was prepared and assigned with a unique identification number based on the clinic identification (ID) code. The selected adolescents were then contacted on the phone, screened for eligibility, and informed about the research objectives as well as place, date, and time of the interview by local study coordinators. Potential participants were included if they were aged between 15 and 17 years, receiving HIV care and treatment services from the selected ART clinics, able to communicate in Khmer, allowed by a parent or guardian to participate, able to present themselves on the day of the interview, and physically and mentally stable to assent and participate in the study. Recruitment continued until the required sample size for each cluster was achieved.

### Variables and measurements

In reference to existing literature [29, 30], a structured questionnaire was developed. Socio-demographic characteristics included age, gender, schooling-related information, parental and caregiver information, and social support the adolescents or their family had received for health care. Clinical and immunological data were obtained from medical records at the clinic. These included CD4 count and viral load at the enrolment in the clinic and the most recent test, duration of receiving HIV care and treatment, type of ART clinic (pediatric or adult), current antiretroviral (ARV) regimen (first or second line), as well as ARV supply and arrangement. We also collected information on adolescents’ preference, experience, and understanding in HIV care and treatment, HIV knowledge, coping strategies, and the maturity of the adolescents in dealing with HIV care and treatment.

### Data collection training

Two teams of interviewers and coordinators were trained for two days on the study protocol, research ethics, confidentiality, and interview techniques. Tool pretesting was conducted among 20 adolescents living with HIV in an ART clinic in Phnom Penh, followed by revisions of contents and language based on feedback from the pre-test. Adolescents who participated in the pre-test were not included in the main study.

### Data analyses

Epi Data version 3 (Odense, Denmark) was used for double data entry and coding. Descriptive analyses were conducted to determine proportions for categorical variables and means and standard deviation (SD) or median and interquartile range (IQR) for continuous variables. In accordance with the national guidelines [5], adolescents were categorized as having viral suppression if the viral load count was < 1000 ribonucleic acid (RNA) copies/mL at the latest viral load count, which differs from the definition used in high-income settings [31]. We used Chi square test (or Fisher’s exact test when a cell count was smaller than five) for categorical variables and Student’s t-test or Mann–Whitney Test as appropriate for continuous variables to compare variables among adolescents with and without viral suppression.

To identify factors associated with viral non-suppression, a multivariate logistic regression model was constructed. Age, gender, and all variables associated with viral non-suppression in bivariate analyses at a level of *p*-value < 0.05 were simultaneously included in the model, followed by a backward selection. p-values of < 0.05 were considered statistically significant. SPSS version 22 (IBM Corporation, New York, USA) was used for all statistical analyses.

### Ethics statement

The National Ethics Committee for Health Research, Ministry of Health, in Cambodia approved the study protocol (Ref: 297NECHR). All participants were required to assent to the study, and a written informed consent from a parent or guardian was obtained before data collection. Interviews were conducted in private locations, and confidentiality was strictly protected by removing all personal identifiers from the sampling frame, questionnaires, and field notes. Each participant was assigned a unique code. Researchers and data collectors were carefully trained on ethical issues. All participants were provided with a token of US$5 for transportation and time compensation.

## Results

### Socio-demographic characteristics

Socio-demographic characteristics of participants are shown in Table 1. This study included 328 adolescents living with HIV with a mean age of 15.9 (SD = 0.8); of whom, 48.5% were female. Less than half (42.7%) reported currently living with their biological parents, and 50.9% reported that their mother was alive. More than half (57.0%) reported that their parent was their main caregiver. In general, level of education of their parents was low, with less than half completing high school. Regarding their own education, 60.4% reported currently attending school full time, while 22.9% were in school but with interruption. About one-third (34.8%) reported that their family received social support for their health care, and 29.6% belonged to a patient support group. The majority (68.6%) were receiving HIV care and treatment from a pediatric ART clinic, and 82.6% were on first line ART regimen.

**Table 1:** Socio-demographic characteristics of adolescents living with HIV with and without viral suppression

Socio-demographic characteristics	Total (*n* = 328) *n* (%)	Latest viral load count	
		≥ 1000 RNA copies/mL (*n* = 76)	<1000 RNA copies/mL (*n* = 252)
		*n* (%)	*n* (%)
Age group (in years)			
15	126 (38.4)	36 (47.4)	90 (35.7)
16	107 (32.6)	25 (32.9)	82 (32.5)
17	95 (29.0)	15 (19.7)	80 (31.7)
Male gender	169 (51.5)	38 (50.0)	131 (52.0)
Currently living with			
Parents	140 (42.7)	44 (57.9)	96 (38.1)
Grandparents	49 (14.9)	15 (19.7)	34 (13.5)
Relatives	106 (32.3)	11 (14.5)	95 (37.7)
In an orphanage	27 (8.2)	4 (5.3)	23 (9.1)
Other	6 (1.8)	2 (2.6)	4 (1.6)
Mother is still alive	167 (50.9)	33 (44.3)	134 (53.2)
Mother’s level of formal education			
Lower than high school	97 (29.6)	31 (40.8)	66 (26.2)
High school or higher	61 (18.6)	12 (15.8)	49 (19.4)
No mother/don’t know	170 (51.8)	33 (43.4)	137 (54.4)
Father is still alive	130 (39.6)	34 (44.7)	96 (38.1)
Father’s level of formal education			
Lower than high school	58 (17.7)	21 (27.6)	37 (14.7)
High school or higher	68 (20.7)	13 (17.1)	55 (21.8)
No father/don’t know	202 (61.6)	42 (55.3)	160 (63.5)
Main daily caregiver			
Parent	187 (57.0)	58 (76.3)	129 (51.2)
Grand parent	5 (1.5)	1 (1.3)	4 (4.6)
Sibling	24 (7.3)	3 (3.9)	21 (8.3)
Relatives	100 (30.5)	11 (14.5)	89 (35.3)
Orphanage/NGO staff	12 (3.7)	3 (3.9)	9 (3.6)
Main caregiver’s education lower than high school	54 (27.7)	11 (32.4)	43 (26.7)
Your level of formal education			
Primary school or lower	92 (28.0)	25 (32.9)	67 (26.6)
Secondary school	168 (51.2)	37 (48.7)	131 (52.0)
High school or higher	68 (20.7)	14 (18.4)	54 (21.4)
Currently attending school			
Not currently in school	55 (16.8)	16 (21.1)	39 (15.5)
With disruption	75 (22.9)	18 (23.7)	57 (22.6)
Fulltime	198 (60.4)	42 (55.3)	156 (61.9)
Family received social support	114 (34.8)	23 (30.3)	91 (36.1)
Belonged to a support group	97 (29.6)	16 (21.1)	81 (32.1)
Type of ART site			
Pediatric	225 (68.6)	36 (47.4)	189 (75.0)
Adult	94 (28.7)	36 (47.4)	58 (23.0)
Other	9 (2.7)	4 (5.3)	5 (2.0)
Receiving first line ARV regimen	271 (82.6)	63 (82.9)	208 (82.5)

*ART* antiretroviral therapy, *CI* confidence interval, *HIV* human immunodeficiency virus, *NGO* non-governmental organization

Compared to 15.8% among adolescents aged 17, 28.6% of adolescents aged 15 had a viral load ≥ 1000 RNA copies/mL (*p* = 0.03). Almost one-third (31.4%) of adolescents living a biological parent had a viral load ≥ 1000 RNA copies/mL compared to 10.4% among adolescents living with a relative (*p* < 0.001). Similarly, the proportion of adolescents having a viral load ≥ 1000 RNA copies/mL was significantly higher among adolescents having a parent as the main caregiver compared to that among adolescents having a relative as the main caregiver (31.0% vs. 11.0%, *p* < 0.001). The proportion of adolescents having a viral load ≥ 1000 RNA copies/mL was also significantly higher among adolescents receiving HIV care in an adult clinic compared to that among adolescents receiving HIV care in a pediatric clinic (38.3% vs. 16.0%, *p* < 0.001).

### Medical history

Table 2 shows that, on average, the adolescents had been in HIV care for 10.1 (interquartile range [IQR] = 8.0–11.5) years and received ART for 8.6 (IQR = 6.0–10.6) years. Median CD4 count at the enrolment was 561 (IQR = 274–798) and at the most recent test was 672 (IQR = 483–827). Median viral load at the first test was 628 RNA copies/mL (IQR = 315–3427) and at the most recent test was 215 RNA copies/mL (IQR = 168–2153). Compared to adolescents with viral suppression, adolescents without viral suppression had received care services from an ART clinic (8.9 years vs. 10.4 years, *p* = 0.002) and been on ART (6.9 years vs. 9.2 years, *p* < 0.001) for a significantly shorter duration. Adolescents without viral suppression had significantly lower latest CD4 count (594 vs. 699, *p* < 0.001) and higher first viral load count (7036 RNA copies/mL vs. 312 RNA copies/mL, *p* < 0.001) than adolescents with viral suppression.

**Table 2:** Medical history among adolescents living with HIV with and without viral suppression

Medical history	Total (*n* = 328)	Latest viral load count	
		≥ 1000 RNA copies/mL (*n* = 76)	< 1000 RNA copies/mL (*n* = 252)
Time since first ART clinic visit (in years)	10.1 (8.0–11.5)	8.9 (5.2–10.4)	10.4 (8.8–11.7)
Time since ART initiation (in years)	8.6 (6.0–10.6)	6.9 (0.1–12.5)	9.2 (1.2–14.3)
CD4 count at enrolment	561 (274–798)	567 (271–710)	561 (277–825)
Latest CD4 count	672 (483–827)	594 (376–723)	699 (529–858)
First viral load count (RNA copies/mL)	628 (315–34,427)	7036 (426–93,625)	312 (218–630)

Values are median (interquartile range)

*ARV* antiretroviral, *ART* antiretroviral therapy, *HIV* human immunodeficiency virus, *RNA* ribonucleic acid

### HIV knowledge

As shown in Table 3, HIV knowledge among adolescents living with HIV in this study was generally good, although room remains for improvement. For example, 42.1% of the participants agreed that there is a cure for AIDS. A considerable proportion of them believed that, if both partners are HIV-positive, they do not have to use a condom (38.1%); if people living with HIV take medicines, they do not have to use a condom (26.2%), and it is okay for people living with HIV to miss their medicines (13.1%). The proportion of adolescents having a viral load ≥ 1000 RNA copies/mL was significantly higher among adolescents who believed that there is a cure for AIDS compared to adolescents who did not believe this (33.3% vs. 13.9%, *p* < 0.001).

**Table 3:** Assessment of HIV knowledge among adolescents living with HIV with and without viral suppression

HIV knowledge	Total (*n* = 328) *n* (%)	Latest viral load count	
		≥ 1000 RNA copies/mL (*n* = 76)	< 1000 RNA copies/mL (*n* = 252)
		*n* (%)	*n* (%)
HIV is a virus that attacks a person’s immune system			
True	263 (80.2)	58 (76.3)	205 (81.3)
False	44 (13.4)	11 (14.5)	33 (13.1)
Don’t know	21 (6.4)	7 (9.2)	14 (5.6)
The cells of immune system that fight infections are called CD4 cells			
True	203 (61.9)	44 (57.9)	159 (63.1)
False	22 (6.7)	4 (5.3)	18 (7.1)
Don’t know	103 (31.4)	28 (36.8)	75 (29.8)
The amount of HIV in a person’s blood is called viral load			
True	160 (48.8)	35 (46.1)	125 (49.6)
False	26 (7.9)	5 (6.6)	21 (8.3)
Don’t know	142 (43.3)	36 (47.4)	106 (42.1)
There is a cure for AIDS			
True	138 (42.1)	46 (60.5)	92 (36.5)
False	165 (50.3)	23 (30.3)	142 (56.3)
Don’t know	25 (27.6)	7 (9.2)	18 (7.1)
You can tell a person has HIV by looking at him or her			
True	46 (14.0)	11 (14.5)	35 (13.9)
False	269 (82.0)	63 (82.9)	206 (81.7)
Don’t know	13 (4.0)	2 (2.6)	11 (4.0)
A person will stay healthy if their CD4 cells are high and their viral load is low			
True	284 (86.6)	68 (89.5)	216 (85.7)
False	23 (7.0)	4 (5.3)	19 (7.5)
Don’t know	21 (6.4)	4 (5.3)	17 (6.7)
Even though a person with HIV may feel healthy, the virus can still be damaging his or her immune system			
True	248 (75.9)	64 (84.2)	184 (73.0)
False	65 (19.8)	10 (13.2)	55 (21.8)
Don’t know	15 (4.6)	2 (2.6)	13 (5.2)
If both partners are HIV positive, they don’t have to use a condom			
True	125 (38.1)	34 (44.7)	91 (36.1)
False	155 (47.3)	33 (44.3)	122 (48.4)
Don’t know	48 (14.6)	9 (11.8)	39 (15.5)
If people living with HIV take medicines, they don’t have to use a condom			
True	86 (26.2)	20 (26.3)	66 (26.2)
False	193 (58.8)	42 (55.3)	151 (59.9)
Don’t know	49 (14.9)	14 (18.4)	35 (13.9)
It is okay for people living with HIV to miss their medicines			
True	43 (13.1)	13 (17.1)	30 (11.9)
False	274 (83.5)	61 (80.3)	213 (84.5)
Don’t know	11 (3.4)	2 (2.6)	9 (3.6)

*CI* confidence interval, *HIV* human immunodeficiency virus, *OR* odds ratio

### Understanding of HIV care and treatment

Table 4 shows that the majority (68.0%) of participants preferred to receive HIV care and treatment services from a pediatric ART clinic; 47.9% preferred to discuss questions related to health, sexual life, or daily life with their family members; 72.0% could recognize when they are getting sick; 88.7% were somewhat prepared or very prepared to manage their treatment going forward; 80.5% knew when they need to call a doctor; and 54.0% were responsible for refilling their own medicines. Regarding adherence to ART, 23.8% found it difficult to remember to take medicines; 11.0% would stop taking medicines when they feel worse; and 14.0% had missed medicines in the past 4 days. The proportion of adolescents having a viral load ≥ 1000 RNA copies/mL was significantly higher among adolescents who responded that they preferred to discuss issues related to health, sexual life, or daily life with their family members compared to adolescents who preferred to discuss these issues with health care providers (29.9% vs. 17.9%, *p* = 0.02).

**Table 4:** Understanding of HIV care and treatment among adolescents living with HIV with and without viral suppression

Understanding in HIV care and treatment	Total (*n* = 328) *n* (%)	Latest viral load count	
		≥ 1000 RNA copies/mL (*n* = 76)	< 1000 RNA copies/mL (*n* = 252)
		*n* (%)	*n* (%)
Preference in receiving HIV care and treatment services			
Pediatric ART clinic	217 (66.2)	44 (57.9)	173 (68.7)
Adult ART clinic	111 (38.8)	32 (42.1)	79 (31.3)
Preferred to discuss questions related to health, sexual life, or daily life with			
Health care providers	67 (20.4)	12 (15.8)	55 (21.8)
Counselors/peer educators	19 (5.8)	5 (6.6)	14 (5.6)
Friends	44 (13.4)	4 (5.3)	40 (15.9)
Family	157 (47.9)	47 (61.8)	110 (43.7)
Other	41 (12.5)	8 (10.5)	33 (13.1)
Could recognize when you are sick	236 (72.0)	177 (70.2)	59 (77.6)
Preparedness to manage your treatment going forward			
Very prepared	42 (12.8)	9 (11.8)	33 (13.1)
Somewhat prepared	249 (75.9)	57 (75.0)	192 (76.2)
Somewhat unprepared	21 (6.4)	9 (11.8)	12 (4.8)
Very unprepared	16 (4.9)	1 (1.3)	15 (6.0)
Know when you need to call a doctor	264 (80.5)	58 (76.3)	206 (81.7)
Responsible for making appointments	151 (46.0)	28 (36.8)	123 (48.8)
Responsible for refilling own medicines	177 (54.0)	35 (46.1)	142 (56.3)
Feel comfortable asking questions	44 (13.4)	10 (13.2)	34 (13.5)
Difficult to remember to take medicines	78 (23.8)	12 (15.8)	66 (26.2)
Stop taking medicines when you feel better	2 (0.6)	0 (0.0)	2 (0.8)
Stop taking medicines when you feel worse	36 (11.0)	12 (15.8)	24 (9.5)
Have missed medicines in the past 4 days	46 (14.0)	13 (17.1)	33 (13.1)

*ART* antiretroviral therapy, *HIV* human immunodeficiency virus

### Factors associated with viral non-suppression

Table 5 shows factors associated with viral non-suppression in a multivariate logistic regression model. After controlling for the effect of other covariates, the likelihood of having viral non-suppression remained significantly lower among adolescents who were aged 17 (AOR = 0.46, 95% CI 0.21–0.98), had been on ART for more than nine years (AOR = 0.35, 95% CI 0.19–0.64), had most recent CD4 count of > 672 (AOR = 0.47, 95% CI 0.26–0.86), had a relative as the main caregiver (AOR = 0.37, 95% CI 0.17–0.80), and did not believe that there is a cure for AIDS (AOR = 0.40, 95% CI 0.21–0.75) compared to their reference group. The likelihood of having viral non-suppression remained significantly higher among adolescents who had first viral load > 628 RNA copies/mL (AOR = 1.81, 95% CI 1.05–4.08) and were receiving HIV care and treatment from an adult ART clinic (AOR = 2.95, 95% CI 1.56–5.59).

**Table 5:** Factors associated with viral non-suppression among adolescents living with HIV (*n* = 328)

Variables in the model	Viral non-suppression^a^			
	OR (95% CI)	*p*-value	AOR (95% CI)	*p*-value^†^
Age group				
15	Reference		Reference	
16	0.76 (0.42–1.38)	0.37	0.80 (0.40–1.57)	0.51
17	0.47 (0.24–0.92)	0.03	0.46 (0.21–0.98)	0.04
Duration on ART^†^ (years)				
≤ 9	Reference		Reference	
> 9	0.26 (0.15–0.45)	< 0.001	0.35 (0.19–0.64)	0.001
Most recent CD4 count^†^				
≤ 672	Reference		Reference	
> 672	0.45 (0.26–0.77)	0.003	0.47 (0.26–0.86)	0.02
First viral load^†^				
≤ 628 RNA copies/mL	Reference		Reference	
> 628 RNA copies/mL	2.04 (1.04–2.50)	0.02	1.92 (1.02–4.42)	0.04
Main daily care giver				
Parent	Reference		Reference	
Grand parent	0.56 (0.06–5.08)	0.60	0.72 (0.06–6.10)	0.80
Sibling	0.32 (0.09–0.11)	0.07	0.30 (0.07–1.18)	0.08
Relatives	0.28 (0.14–0.55)	< 0.001	0.37 (0.17–0.80)	0.01
Orphanage/NGO staff	0.74 (0.19–2.84)	0.66	0.49 (0.10–2.32)	0.37
Receiving HIV care and treatment from:				
Pediatric ART clinic	Reference		Reference	
Adult ART clinic	3.26 (1.88–5.64)	< 0.001	2.95 (1.56–5.59)	0.001
There is a cure for AIDS				
True	Reference		Reference	
False	0.32 (0.18–0.57)	< 0.001	0.40 (0.21–0.75)	0.005
Receiving HIV care and treatment from:				
Pediatric ART clinic	Reference		Reference	
Adult ART clinic	3.26 (1.88–5.64)	< 0.001	2.95 (1.56–5.59)	0.001

Age, gender, and all variables associated with viral non-suppression at a level of p < 0.05 were simultaneously included in a multivariate logistic regression model

*AOR* adjusted odds ratio, *ART* antiretroviral therapy, *CI* confidence interval

^a^Viral load count of < 1000 RNA copies/mL

^†^Median value was used as a cutoff to categorize the variables

## Discussion

After an average of 8.6 years on ART, the rate of viral suppression among adolescents living with HIV in this study was considerably high at 76.8%. This observed level of viral suppression falls short of the UNAIDS's 90–90–90 global targets, but it is well within the range reported in other studies. In a recent review, eight studies reported that the proportion of adolescents with viral suppression at 12 months ranged from 27 to 89% [32]. Another review that included 11 cohort studies also reported viral suppression rates between 28 and 78% [3]. Apart from reporting the proportion of adolescents with suppressed viral loads, the present study also identified independent predictors of viral non-suppression, which have important implications for HIV clinical service delivery across the study sites.

Older age may predict viral suppression. This might be related to the increasing treatment experience among older long-term survivors as reported in other studies [33, 34]. In Cambodia, treatment regimen and formulations for people living with HIV aged over 14 years old are similar to that for adults as long as they weigh more than 35 kg [5]. Because adolescence is characterized by increasing cognition [35–37], the relationship between viral suppression and age among long-term survivors of HIV could be mediated by the impact of self-efficacy and self-competency and risk-taking on ART adherence [3, 33]. Similar findings regarding age were also found in a study among people living with HIV in South Africa, whereby adolescents aged < 15 were more likely to have unsuppressed viral load (viral load > 400 RNA copies/mL) compared with older patients [38]. Indeed, studies have consistently reported that increasing age reduced odds of non-viral suppression in Uganda (≥ 1000 RNA copies/mL) [39] and in several countries across Europe (> 50 RNA copies/mL) [40].

Shorter duration on ART was associated with viral non-suppression, which is to be expected given the destruction of CD4 cells resulting from high viral replication. Although the relationship between viral non-suppression, immunological responses, and duration on ART is not always consistent [41], similar findings were reported in a South African study, which reported that adolescents who had been on ART between six and 12 months were more likely to have viral non-suppression (viral load > 400 RNA copies/mL) compared with those who had been on treatment longer [38]. In our study, a lower viral load at the time of initial clinic enrollment also predicted viral suppression, which is in line with mechanisms of ART action [3].

Preference for receiving HIV care and treatment from a pediatric ART clinic predicted viral suppression. This could be because of the existing familiarity and ability of pediatric providers to provide appropriate counseling to young people in Cambodia. According to the national monitoring data, the majority of the Cambodian adolescents living with HIV are perinatally infected [5], and this may lead to having close relationships and contacts with their pediatric providers. Previous studies in Cambodia have shown that adolescents highly value current relationships with pediatric health providers and do not trust adult providers to maintain confidentiality [24, 25].

We also found that the belief that there is no cure for AIDS predicted viral suppression. This could be an indication of the perception of the importance of ART, and potentially the importance of adherence. It is also possible that this is a reflection of appropriate education and counseling, which may also reinforce adherence. Poor treatment literacy and comprehension of the benefits of ART as a curative intervention negatively affects adherence [42].

Several implications emanate from findings in this study. First, better evaluation of adherence among the 24.2% who were not virally suppressed is warranted. Second, practical interventions are needed to strengthen adherence among this subsample. These interventions need to be tailored to the individual needs of each adolescent, and a combination of multiple approaches may be necessary. For instance, focusing particularly on younger adolescents and adolescents in transition to adult clinic with a range of counseling and social support could improve their treatment outcomes. Data from this sample (published elsewhere) suggest that transition to adult clinic is an ongoing challenge [23].

Data from this study showed that more than 88.0% of the adolescents were somewhat prepared or very prepared to manage their own treatment, which reflects the strong self-preparedness and self-reliance for transition. One of the challenges encountered for transition is the preparedness at the adult provider side. This could have an implication on the observation that  68.0% of adolescents preferred to receive services at a pediatric ART clinic. Similarly, enhancing counseling and treatment literacy to improve adolescents’ understanding of the importance of ART and the currently incurable nature of HIV could improve adherence to the treatment and viral suppression. For younger adolescents, the education and information could be reinforced with their main caregivers and themselves during routine patient support group sessions.

### Study limitations

This study has a number of limitations, which should be taken into account while interpreting the above findings. First, due to the sampling strategy, the study only included adolescents who were currently on treatment. Adolescents who were lost to follow-up were not included, which could have resulted in overestimating the rate of viral suppression. Longitudinal follow up with multiple assessments of viral load trends may provide a better picture of virological response compared to the single latest viral load measurement used in this study. Second, the study relied on self-reported data, which may be affected by recall and social desirability bias [43]. Third, adolescents who accessed care from low volume sites providing care to less than 10 adolescents were excluded, which may bias the results given that volume of patients is a key determinant of provider competency [44] and patient outcomes [45]. Finally, due to a lack of genotype data for the sample, exploration of virological failure was not possible.

## Conclusions

The proportion of adolescents living with HIV in this study who had viral load < 1000 RNA copies/mL was relatively high at 76.8%. However, this proportion falls short of the UNAIDS’ 90% target for on-treatment viral suppression. Findings from this study indicate that programs targeting younger adolescents and adolescents in transition from pediatric to adult clinics with a range of interventions including psychosocial support and treatment counseling could further improve viral suppression outcomes among this young population.
